# Uniquely altered transcripts are associated with immune preservation in HIV infection

**DOI:** 10.1371/journal.pone.0169868

**Published:** 2017-03-28

**Authors:** Michelle Zanoni, Ítalo Karmann Aventurato, James Hunter, Maria Cecilia Araripe Sucupira, Ricardo Sobhie Diaz

**Affiliations:** Retrovirology Laboratory, Department of Medicine, Federal University of Sao Paulo, Sao Paulo, Brazil; Universita Vita Salute San Raffaele, ITALY

## Abstract

The mechanisms underlying host HIV control hold much promise in the search for a functional HIV cure. We investigated the host genomic signatures in elite controllers or rapid progressors following recent infection and the correlates of immune reconstitution during combination antiretroviral therapy. We characterized the HIV-specific longitudinal host transcriptional response of peripheral blood mononuclear cells from elite controllers, rapid progressors, immune responders and non-responders using a RT-qPCR array in a cohort of recently HIV-infected Brazilian individuals. The elite controllers expressed unique transcripts early in infection that were closely associated with specialized cross-presentation between XCR1^+^ DCs and antigen-specific CD8^+^ T cells (XCL1). The natural suppression of HIV was also associated with the highly functional co-expression of cytokines and chemokines (CCL2, TNF and IL-10) concomitant with the maintenance of important anti-inflammatory and anticoagulant properties (Antithrombin III). Immune responders exhibited exclusively upregulated mRNAs possibly related to stem cell mobilization before combination antiretroviral therapy (neutrophil elastase). Our longitudinal approach to gene expression permitted us to discover previously unrecognized determinants that contribute to natural or antiretroviral-mediated HIV-1 immune control.

## Introduction

Whereas there is a growing body of evidence recognizing the importance of host responses in HIV infection outcomes [1], understanding how the host can control the virus may guide novel therapeutic strategies to reverse latency and cure the infection in patients on combination antiretroviral therapy (cART), which is a major challenge. Host responses to cART can also be heterogeneous despite achieving viral suppression and can sometimes include a lack of proper CD4^+^ T cell reconstitution [2].

Although robust cytotoxic T lymphocyte (CTL) and attenuated viral fitness are associated with spontaneous immune HIV control [3, 4], several other host genetic and immunologic events have been described, such as an over-representation of “protective” HLA class I alleles, including HLA-B*57, HLA-B*27, HLA-B*13 and HLA-B*58:01 [5, 6] and a higher production of granzyme, perforin and granulysins by HIV-1-specific CD8^+^ T cells [7–9].

There is still a large discrepancy between the life expectancy of the general population and the life expectancy of an HIV-infected individual, who can experience different degrees of ‘damage-response’ framework [10] related to the long-term persistence of HIV, even with cART [11]. Most critical are the individuals whose CD4^+^ T cell recovery remains impaired despite virological suppression, which has been closely connected with several risk factors (*e*.*g*., increased immune activation and poor bone marrow function [12, 13]) and with an increased risk of adverse outcomes, including serious non-AIDS events [13, 14].

To address the mechanisms underlying host HIV control and immune reconstitution during effective cART, we focused on identifying a common host transcriptional response to HIV infection in a cohort of recently HIV-infected Brazilian individuals. We hypothesized that a longitudinal dissection of the gene expression that is unique to elite controllers during the earliest stages of infection as well as the gene expression of individuals treated with cART might help us better understand the host factors that contribute to the immune control of HIV-1.

## Materials and methods

### Study subjects

Peripheral blood mononuclear cells (PBMCs) were obtained from 20 HIV-infected individuals enrolled in the recent HIV infection cohort in the city of Sao Paulo, Brazil [15, 16] and from 5 healthy HIV non-infected individuals. The HIV-infected individuals met the criteria to represent four outcomes, with five subjects per group: elite controllers, rapid progressors, immune responders and immune non-responders. All subjects provided written informed consent to participate in this study under protocols approved by the Federal University of Sao Paulo Human Research Ethics Committee (1586/11_ID), and Ethics committee approve this consent procedure. Written informed consents are kept in a safe cabinet at the University of Sao Paulo, Sao Paulo, Brazil, under the guard of Professor Esper Kallas, the Principal Investigator of this cohort of patients.

#### Recent infection determination

Recent infection was defined as a nonreactive detuned HIV-1 antibody test consistent with infection for less than six months at the time of screening, or documented seroconversion (*i*.*e*., a documented negative HIV-1 enzyme immunoassay [EIA] or a negative or indeterminate Western blot within six months before the study). A Vironostika HIV-1 Microelisa System (BioMerieux, Raleigh, NC, USA) EIA was performed as a recent infection determination assay, with a recent infection probably acquired in the last 170 days (95% confidence interval: 145–200 days) defined as a standardized optical density measurement ≤1.0. All recently HIV-infected individuals had fully seroconverted during follow-up after recruitment.

#### cART-naïve recent HIV-infected subjects

**Fig 1:**
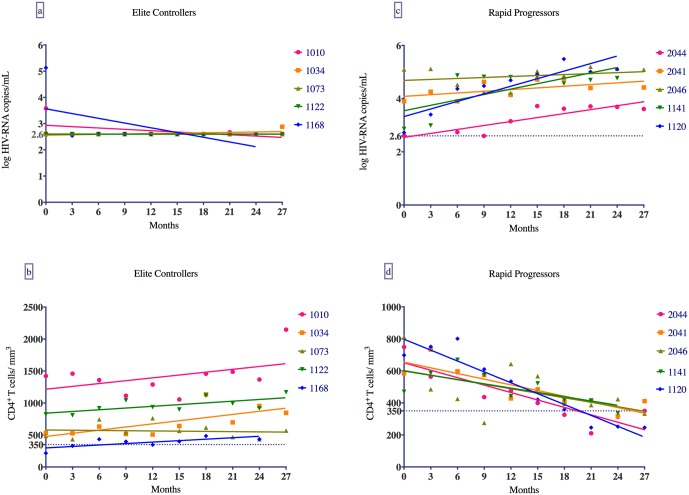
Linear regression graphs of viral loads and CD4^+^ T cell counts over 24 months. Elite controllers (panels a and b) and rapid progressors (panels c and d). Each dot represents data from a single recently HIV-infected individual analyzed every 3 months immediately after cohort enrollment.

Due to the variability of definitions and follow-up times in the elite controller and rapid progressor cohorts, a distinct pace of disease progression was determined based on virological and immunological criteria. Five cART-naïve recent HIV-infected individuals defined as elite controllers and five defined as rapid progressors were assessed based on the opposite slopes of their CD4^+^ T cell counts and viral loads over 24 months, which were retrospectively analyzed every 3 months immediately after enrollment (Fig 1a–1d).

HIV positive elite controllers were selected based on having consecutive viral loads < 40 copies/mL after confirmed recent infection over 24 months, varying from 21 to 34 months of undetectable viral loads (median of 29 months and mean of 28 months), with a median of 10 and a mean of 9 undetectable viral loads/subject, in the absence of viral blips. Initial bursts of viremia of 3,800 copies/mL and 136,079 copies/mL were recorded in two elite controllers during the early stage of infection (Fig 1a). To examine changes in the CD4^+^ T cell count over time, a linear regression analysis was generated and the highest values for slope (β, coefficient) over 24 months following recent infection were selected. A negative slope was recorded in one subject—that is, the count declined—because all CD4^+^ T cell counts were above 500 cells/mm^3^ over the 24 months (Fig 1b). Rapid progressors were selected based on linear regression analysis of their viral loads and CD4^+^ T cell counts during the course of infection. Individuals with the highest viral loads and the lowest CD4^+^ T cell counts over 24 months following recent HIV infection were selected (Fig 1c and 1d). The mean and median time for rapid progressors to evolve to a CD4^+^ T cell counts to a level below 350 cells/mm^3^ was 27 and 27 months respectively. First mean and median viral loads for rapid progressors during the recent infection period was 3.4 log and 2.9 log respectively, and mean and median viral loads after 1 year of follow up (basal viral loads) was 4.2 log and 4.2 log respectively. All rapid progressors were antiretroviral treated upon CD4^+^ T cell counts decreased to a level below 350 cells/mm^3^, but samples were analyzed here during the antiretroviral naïve period for this group of individuals.

#### cART-suppressed chronic HIV-infected subjects

Due to the heterogeneity in host responses to cART despite achieving complete suppression of the viral load, CD4^+^ T cell reconstitution was evaluated in two groups of cART-suppressed chronic HIV-infected subjects. One of the groups was comprised of five immunological responders who presented consecutive viral loads of < 400 copies/mL in the absence of blips during the first 12 months of HIV suppressive cART. To examine CD4^+^ T cells count changes in relation to the evolution of the infection, a linear regression model was generated and the individuals with the highest β coefficients over 12 months following cART suppression were selected (Table 1). The other group was comprised of five immunological non-responders selected according to previous definitions [2], who presented consecutive viral loads of < 400 copies/mL in the absence of blips during the first 12 months of suppressive cART and an increase in the CD4^+^ T cell counts of < 30% during the first 12 months of HIV suppressive cART. An increase in the CD4^+^ T cell counts of > 30% was observed in one subject who failed to acquire an absolute CD4^+^ T cell count < 200 cells/mm^3^ during the first 12 months of viral suppression in the immunological non-responders’ group (Table 1). cART-suppressed chronic HIV-infected subjects were homogeneously treated with a regimen comprised of an association of fixed dose combination of zidovudine and 3TC administered BID, and a QD dose of Efavirenz, according to the local Brazilian guidelines at that time.

**Table 1:** Characteristics of cART suppressed chronic HIV-infected patients.

	Immune responders					Immune non-responders				
Patient ID	1046	1087	1117	2028	2033	1050	1097	2016	2020	2042
VL, before cART (copies/mL)	222000	2730	183000	506000	40500	11500	106000	62900	9330	12100
VL, 12 months after cART (copies/mL)	<400	<400	<400	<400	<400	<400	<400	<400	<400	<400
CD4, before cART (cells/mm^3^)	268	255	261	227	235	240	83	375	143	255
CD4, 12 months after cART (cells/mm3)	628	533	496	503	450	296	185	331	112	287
Increase in CD4 during 12 months (%)	134	109	90	122	91	23	123	-12	-22	13

VL indicates viral load. The mean time of the earliest available PBMC sample before cART used for gene expression analysis was 4 months for immune responders and 2 months for non-responders.

### HLA class I typing, HIV subtype assignment and co-receptor tropism use

The subjects were typed for major histocompatibility complex (MHC) class I antigen expression with intermediate resolution using sequence-specific primer PCR kits (Pel-Freez SSP UniTray; Invitrogen, Carlsbad, CA) according to the manufacturer’s instructions. HIV subtype assignment and co-receptor tropism prediction according to the gp120 V3 region DNA sequencing were performed as previously described. [16]

### Gene expression profiling and data analysis

To investigate how gene expression patterns changed over time, PBMCs were assayed from each subject across two time points of infection. For the cART-naïve recent HIV-infected group, time point 1 was the earliest available sample during recent HIV infection and time point 2 was 12 months after the first time point. For the cART-suppressed chronic HIV-infected group, time point 1 was the first available sample before treatment and time point 2 was approximately 12 months after the initiation of cART. All cART was initiated immediately after the CD4^+^ T cell counts decreased to a level below 350 cells/mm^3^. The relative composition of CD4^+^ T cells, CD8^+^ T cells and monocytes of the bulk PBMC population analyzed have not been analyzed.

Total RNA was isolated from (5–10) x 10^6^ PBMCs using the RNeasy Mini Kit (cat. no. 74104; Qiagen) with on-column DNase treatment (RNase-Free DNase Set, Qiagen [cat. no. 79254]) according to the manufacturer’s instructions. The RNA purity and integrity were assessed using a NanoDrop ND-1000 spectrophotometer and a lab-on-chip Agilent RNA 6000 Nano Kit (Bioanalyzer 2100), respectively, according to Fleige and Pfaffl (2006). One microgram of RNA was reverse transcribed into cDNA using the RT2 First Strand Kit (cat. no. 330401; SABiosciences) according to the manufacturer’s instructions. Quantitative real-time PCR was assayed using a Standard SYBR Green 96-well plate PCR Array—Human HIV Infection and Host Response (cat. no. PAHS-051A; SABiosciences) following the manufacturer’s instructions. Controls including genomic DNA, reverse-transcription and PCR controls were performed in replicates to test for inter-well and intra-plate consistency. The *B2M* (Beta-2-microglobulin), *HPRT1* (Hypoxanthine phosphoribosyltransferase 1), *RPL13A* (Ribosomal protein L13a), *GAPDH* (Glyceraldehyde-3-phosphate dehydrogenase) and *ACTB* (Actin, beta) housekeeping genes included in the arrays enabled the normalization of the data using the Bestkeeper algorithm [17]. The same threshold value was set across all 45 samples, and the fold change was calculated by the relative gene expression method determined using the equation 2^-ΔΔCT^, as previously described in the literature [18]. The results were considered statistically significant if p ≤ 0.05 as determined by Student’s t-test, as implemented in the SABiosciences Data Analysis package: (http://pcrdataanalysis.sabiosciences.com/pcr/arrayanalysis.php). Differentially expressed genes were defined as those with at least a 2-fold change in mRNA in the HIV-infected compared with the uninfected controls, with p ≤ 0.05. All raw data, programs (in the R language) to process that data, figures and tables have been deposited in a publicly available repository: https://github.com/jameshunterbr/HostTransNetwork. They are available without restriction for consultation and use.

## Results

### HLA class I typing

As seen in Table 2, HLA B*57 was detected in 3 out of the 5 elite controllers and was not detected in the other groups (Fisher’s exact test, p < 0.005). Differences in the gene expression among the elite controllers that had HLA B*57 compared to those without HLA B*57 included a significant threefold upregulation of chemokine (C-C Motif) receptor 4 (CCR4) (p = 0.03) and a threefold upregulation of complement component receptor 2 (CR2) (p = 0.01); the former is selectively expressed on the majority of Th2 cells in adult peripheral blood [19], and the latter has been shown to play a key role in the humoral immune response [20]. There was also a 1.8-fold upregulation of *ELA2*, which is involved in the egress of progenitor cells from the bone marrow [21].

**Table 2:** HLA class I typing according to distinct phenotypes of the response to HIV-1 infection.

	Patient ID	HLAB*	Protective HLA (B*57, B*27, B*13 and B*58:01)	HIV Subtype
Elite controllers	1010	B*39,B*57	Yes	ND
	1034	B*15, B*57	Yes	ND
	1073	B*35, B*57	Yes	ND
	1122	B*51,-	No	B
	1168	B*35,B*40	No	F
Rapid progressors	1120	B*15,B*44	No	B
	1141	B*45,B*52	No	F
	2041	B*35,B*50	No	B
	2044			B
	2046	B*15,B*35	No	B
Immune responders	1046	B*15, B*39	No	B
	1087	B*08,B*52	No	ND
	1117	B*08,B*41	No	F
	2028	B*35,B*45	No	B
	2033	B*07,B*58	Yes	B
Immune non-responders	1050	B*15, B*27	Yes	B
	1097	B*44,B*58	Yes	B
	2016	B*35,B*44	No	C
	2020	B*07,B*44	No	B
	2042	B*15,B*39	No	B

Protective profiles are indicated, with HLA B*57 being more prevalent among elite controllers (Fisher’s exact test, p < 0.005). HIV-1 subtype is also indicated, according to the C2-V3-C3 region profile of the gp120 *env* gene. [16] (ND: not done due to the failure of PCR amplification).

### Distinctive gene expression patterns characterize cART-naïve and cART-suppressed groups over the course of HIV infection

Based on the extensive examination of the published literature, all statistically significant fold changes (p ≤ 0.05) were grouped into functionally similar categories, as shown in Table 3. Immune non-responders were strikingly distinguishable from uninfected subjects, with the highest percentage of altered transcripts (30%) after 12 months of suppressive cART. As is depicted in Table 3, the cART-naïve and cART-suppressed groups differed over the course of HIV infection compared with uninfected controls both quantitatively (the percentage of genes differentially expressed) and qualitatively (functional similarities of the genes differentially expressed). Globally, with the exception of the downregulation of *CD4*, *CCR4* and *RBL2*, most of the altered transcripts increased in expression.

**Table 3:** Mean fold changes in mRNA from HIV-infected individuals compared with uninfected controls.

	cART-naïve				cART-suppressed			
Gene functional classification	Elite controllers		Rapid progressors		Immune responders		Immune non-responders	
	Recent Infection	12 months after infection	Recent Infection	12 months after infection	Before cART	12 months after cART	Before cART	12 months after cART
Apoptosis			***CASP3*** 2.0	***CASP3*** 2.0		*CASP3* 1.7		*CASP3* 1.9
	***CASP8*** 2.1		***CASP8*** 2.4					
				***TRAIL*** 2.4			*TRAIL* 1.6	
				***TNFR2*** 2.4			***TNFR2*** 2.4	***TNFR2*** 2.3
	*ASK1* 1.6	*ASK1* 1.7				*ASK1* 1.5		
Acute Response				*SERPINA1* 1.8	*SERPINA1* 1.6			*SERPINA1* 1.7
	***ATIII*** 3.4	***ATIII*** 2.3						
					***ELA2*** 3.2			
					***SLPI*** 2.8			
			*MBL2* 1.9					***MBL2*** 2.0
Chemokine Receptors	***CCL2*** 6.9							***CCL2*** 27.0
	*CX3CL1* 1.8							***CX3CL1*** 2.0
	***XCL1*** 4.8							
		***CCL8*** 3.0					***CCL8*** 5.4	***CCL8*** 7.5
			CCR4* -1.5	CCR4* -1.9				CCR4* -1.6
		***CXCL12*** 2.2	***CXCL12*** 2.0	***CXCL12*** 3.8				***CXCL12*** 2.4
CDs								***CD209*** 2.5
	CD4* -1.6				CD4* -1.9			
						*CD44* 1.5		
Cytokines	***TNF*** 5.1				***TNF*** 3.1			
								***IL12B*** 2.2
								***IL1B*** 12.7
	***IL10*** 3.6						***IL10*** 15.2	***IL10*** 11.3
								*TGFB1* 1.7
Virus Budding	***TSG101*** 2.1		*TSG101* 1.9	*TSG101* 1.9	*TSG101* 1.7	*TSG101* 1.8	*TSG101* 1.6	***TSG101*** 2.3
Innate Immune Response			***IFNA1*** 2.1					***IFNA1*** 2.2
			***IFNG*** 3.4					
Modulation and Differentiation		***STAT1*** 2.4	*STAT1* 1.9	***STAT1*** 3.2	***STAT1*** 3.0	***STAT1*** 2.1	***STAT1*** 3.1	
		*STAT3* 1.3				*STAT3* 1.3		*STAT3* 1.3
Cell Activation. Transcription and Proliferation								
Proliferation Inhibitors	***CDKN1A*** 2.6			***CDKN1A*** 2.7	***CDKN1A*** 3.7			***CDKN1A*** 4.9
				RBL2* -1.7	RBL2* -2.0			RBL2* -2.0
Transcriptional Activation and Elongation	*CDK7* 1.7		*CDK7* 1.7	*CDK7* 1.6	*CDK7* 1.5			*CDK7* 1.7
				*CDK9* 1.5				*CDK9* 1.6
				*CCNT1* 1.6				*CCNT1* 1.6
Transcriptional Coativators				*EP300* 1.6				
				***CEBPB*** 2.0			***CEBPB*** 2.6	***CEBPB*** 3.3
AP-1 and NF-κB Transcriptional Activation		***FOS*** 3.8			***FOS*** 7.2	***FOS*** 8.1		***FOS*** 8.3
		*LTBR* 1.4		*LTBR* 1.6	*LTBR* 1.5	*LTBR* 1.5		***LTBR*** 2.2

The table shows only genes with statistically significance at the 0.05 level between the two groups. The groups and different time points studied are depicted as columns. All genes are expressed at higher levels than the control, and the fold change is depicted to the right of each gene. The more differentially expressed genes are highlighted in bold.

*Asterisks indicate downregulated genes.

### Enhanced mRNA expression of a unique set of chemokines and cytokines influences HIV control during early infection

**Fig 2:**
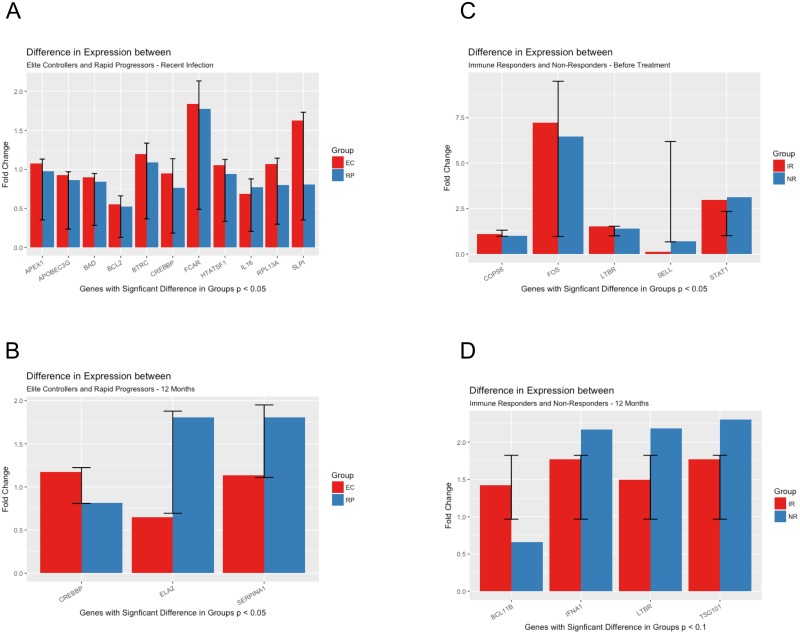
Differences in gene expression between cART-naïve and cART-suppressed groups (p<0.05). (a) Elite Controllers and Rapid Progressors during recent HIV-1 infection. (b) Elite Controllers and Rapid Progressors after 12 months of cohort recruitment. (c) Immunological Responders and Immunological non-responders immediately before antiretroviral treatment initiation. **(d)** Immunological Responders and Immunological non-responders after 12 months of antiretroviral treatment initiation).

To determine the genomic contributors to the host’s capacity to control HIV replication during early infection, we identified differentially expressed genes with a minimum 2-fold change in mRNA in the elite controllers compared to the rapid progressors. The upregulated transcripts restricted to elite controllers early in infection included genes involved in enhanced dendritic cell cross-presentation to antigen-specific CD8^+^ T cells, such as *XCL1*/lymphotactin (mean fold change of 4.8; p = 0.037 for elite controllers), combined with those involved in inhibiting HIV-1 entry into mononuclear cells, such as *CCL2*/monocyte chemoattractant protein-1 (6.9; p = 0.03) and *TNF*/tumor necrosis factor-alpha (5.1; p = 0.005) and those associated with an anti-inflammatory environment, such as *IL10*/interleukin-10 (3.6; p = 0.02). The genes with greatest differences expression between Elite Controllers and Rapid Progressors during recent infection are depicted on Fig 2a.

### Elite controllers have distinct increases in the transcription factor c-Fos and monocyte chemoattractant protein-2 (*CCL8*) upon 12 months of follow-up

After 12 months of follow-up, the upregulated mRNAs in elite controllers included genes involved in *(i)* R5 virus suppression (mean fold change of 2.98 and p = 0.03 for *CCL8*) and *(ii)* the suppression of pro-inflammatory cytokine production (mean fold change of 3.8 and p = 0.04 for *FOS*). The genes with greatest differences expression between Elite Controllers and Rapid Progressors after 12 months of follow up are depicted on Fig 2b.

### Antithrombin III (*ATIII*) expression is associated with durable virus control over time

Throughout the infection period, defense mechanisms may have contributed to spontaneous control of HIV-1 in elite controllers, including the potent anticoagulant activity exerted by high antithrombin III (*ATIII*) expression (mean fold change of 3.4; p = 0.039 for elite controllers in an early stage of infection and 2.3; p = 0.049 in the chronic stage).

### Acute phase proteins and TNF-α may predict immune reconstitution before cART

To predict the genomic determinants of the host’s capacity for successful CD4^+^ T cell recovery, we identified uniquely altered genes in the immune responders compared to the non-responders before cART. The upregulated transcripts restricted to immune responders before treatment included genes involved in the egress of progenitor cells from the bone marrow, as suggested by an increase in *ELA2* (mean fold change of 3.16; p = 0.02), combined with their natural regulators, represented by *SLPI* (mean fold change of 2.82; p = 0.01). High *TNF* expression may indicate the inhibition of HIV-1 entry into mononuclear cells. The genes with greatest differences expression between immunological responders and INR immediately before antiretroviral treatment ignition and after 12 month of antiretroviral treatment are depicted on Fig 2c and 2d.

## Discussion

The ultimate outcomes of HIV-1 infection depend on the initial host-viral interactions that occur early during infection [22, 23]. We therefore chose a cohort of recently HIV-infected individuals in the city of Sao Paulo, Brazil to help delineate the temporal genomic factors that contribute to spontaneous or artificial HIV-1 immune control. We used data from these individuals to evaluate common and specific gene expression patterns in the antiretroviral naïve individuals experiencing a distinct pace of HIV disease progression and individuals experiencing discordant CD4^+^ T cell recovery after cART. This cohort was ideal for these studies because PBMC samples were available following recent infection and because the cART-suppressed chronic HIV-infected subjects were also evaluated from the same well-characterized population under equal conditions.

We are not aware of any data in the literature on the capacity of elite controllers during early infection to express higher levels of XCL1. Here, we provide evidence that PBMCs from elite controllers during early infection produce higher mRNA levels of XCL1 than PBMCs from uninfected subjects. XCL1 is primarily produced by activated CD8^+^ T cells and NK cells [24] and recruits T lymphocytes and dendritic cells by binding to and activating its specific cellular receptor, XCR1 [25, 26]. XCL1 was recently reported to inhibit HIV-1 at an early step of the viral replication cycle via the blockade of viral attachment and entry into host cells [27]. Our results seem to be consistent with a recent observation that described substantial increases in the abundance of mRNAs encoding lymphotactin (XCL1); the antiviral cytokines macrophage inflammatory proteins MIP-1α, MIP-1αP (CCL3L1), and MIP-1β; granulocyte-macrophage colony-stimulating factor (GM-CSF); tumor necrosis factor receptor superfamily member 9 (TNFRSF9); and gamma interferon (IFN-γ) in Gag p24- and Nef-specific CD8^+^ T cells from HIV-1-infected virus controllers (VCs) (less than 5,000 HIV-1 RNA copies/mL and CD4^+^ lymphocyte counts of higher than 400 cells/mm^3^) [28]. The recent demonstration that there is an association between CD8^+^ T cells and primary human CD141^+^ DCs should also be considered. When dendritic cells (DCs) are not directly infected, the debris of cells that were infected and have subsequently undergone apoptosis is taken up and cross-presented by specialized DCs to CD8^+^ T cells. In humans, primary CD141^+^ DCs are the only DC population in the blood that expresses the chemokine receptor XCR1 and responds to the specific ligand XCL1 with Ca^2+^ mobilization and potent chemotaxis. CD141+ DCs excel in the cross-presentation of soluble or cell-associated antigens to CD8^+^ T cells when directly compared with CD1c^+^ DCs, CD16^+^ DCs, and plasmacytoid DCs (pDCs) [29]. In this regard, we suggest that our observation of upregulated XCL1 only in elite controllers during early infection might indicate communication between cross-presenting XCR1^+^DCs and antigen-specific CD8^+^ T cells that should be investigated in future studies. We recognize that the assessment of XCL1 in CD8^+^ cells would be strongly required to validate the above results and confirm our hypothesis, but unfortunately, we do not have further access to biologic material of those patients.

In addition, our transcriptional profiling identified a functional difference between well-documented cytokines and chemokines between elite controllers and rapid progressors during early infection; there was a significant co-expression of *MCP1*, *TNF* and *IL10* only in elite controllers. These findings correspond to studies that show that the functionality of the HIV-specific T-cell response is a clinical correlate of protection from disease progression [30]. Interestingly, in addition to a higher prevalence of HLA B*57 among elite controllers, which has already been described as a protective phenotype against HIV disease progression [5, 6], *CCR4*, *CR2* and *ELA2* were also hyperexpressed among HLA B*57-expressing elite controllers compared to non-HLA B*57 individuals. These data suggest different mechanisms of HIV control among different human hosts.

Importantly, there is also a great need to reduce HIV-induced inflammation and its chronic consequences, including cardiovascular diseases and HIV-related dementia. Here, we present evidence that our cART-naïve patients seem to have achieved one year of control over HIV replication with no decay of CD4^+^ T cells thanks to an exceptional increase in the expression of the chemokine MCP-2 and the transcription factor c-Fos. This is in agreement with previous results showing that MCP-2 is a ligand for CCR5 on CD4^+^ lymphocytes and that it can specifically block R5 HIV-1 [31]. Although data focusing on c-Fos in the pathogenesis of HIV infection are scarce, our current results may be related to the reported capacity of this component of AP-1 to mediate an immunosuppressive effect, acting as an anti-inflammatory transcription factor *in vivo* [32–34]. However, the direct impact of c-Fos on the proper maintenance of such immune regulation deserves further exploration. Concurrently and as a novel finding, we found that the serpin antithrombin III was uniquely upregulated in elite controllers throughout the infection period, which may have had beneficial effects on the limitation of coagulopathic damage [35]. Antithrombin III exerts an anti-inflammatory activity that is mediated in part by the reduction of NF-kappa B activity [36] and, ultimately, the inhibition of HIV-1 replication [37, 38]. Together, these findings indicate that elite controllers are highly equipped to effectively restrain the virus and modulate immune activation starting in the early stages of infection.

We also examined the variability of CD4^+^ T cell recovery to more fully understand the genomic determinants of immunologic efficacy. Remarkably, as a result of these investigations, we found a high level of neutrophil elastase (*ELA2*), which is not expressed by neutrophils, but by their bone marrow precursor cells [21]. As a novel finding as well, we observed *ELA2* transcript levels in PBMCs from immunological responders before cART, which was unexpected and suggests that mechanisms underlying the mobilization of hematopoietic progenitor cells are possibly involved. It has previously been proposed that increased intramedullary elastase cleaves stromal cell vascular cellular adhesion molecule-1 (VCAM-1), reducing cellular adhesiveness and mobilizing marrow cells in the circulatory system [39]. Although our results may eventually predict immune reconstitution following cART, they require further investigation.

It is interesting to note that one of the natural regulators of neutrophil elastase, the secretory leukocyte protease inhibitor (SLPI) [40, 41], was also found to be consistently upregulated only in the same immune responders before cART, confirming its well-documented dose-dependent reciprocal feedback regulation [42–44]. In addition to its antiprotease activity, SLPI has been shown to inhibit NF-kB activation in monocytes [45], exerting anti-inflammatory effects, and to inhibit the ELA2-dependent conversion of proepithelin to epithelin, thereby stimulating wound healing by promoting epithelial cell growth [46]. Furthermore, in the context of susceptibility to HIV infection, high expression of SLPI has been frequently shown to be a protector factor [47]. Taken together, these key effectors of the innate immune system may effectively predict immune reconstitution before cART.

This study has some limitations. First, we investigated a relatively small sample size, and our results need to be confirmed in larger populations of HIV-infected elite controllers, rapid progressors and cART-suppressed individuals. Therefore, the results presented here should be considered preliminary. Second, this study was performed using RNA isolation from bulk PBMCs, and as such, it cannot determine which cells are demonstrating altered gene expression. Therefore, further functional and diagnostic approaches, such as the identification and sorting of elite controller and immunological non-responder cell subsets, ex-vivo infection assays in cell culture or siRNA-mediated gene knockdown, will be important in advancing the findings presented here in future studies. Furthermore, although we applied a systematic comparison across all studied groups, we intentionally chose to explore and discuss only the unique differentially expressed genes (at least a 2-fold change, with p ≤ 0.05) in elite controllers and immune responders due to space limitations. However, this study was able to focus on a non-specific predefined pathway, enabling us to discover previously unrecognized correlates of immune protection and providing insights in the search for a functional cure among HIV-infected individuals.

## Conclusions

Differentially expressed genes drive phenotype variation, and this study solidifies the notion that elite controllers in the early stages of HIV infection have unique genetically programmed immune responses to control initial HIV spread while maintaining anti-inflammatory signaling in peripheral mononuclear cells. We have shown that altered transcripts vary across cART-naïve and cART-suppressed groups over time in the course of HIV infection compared with uninfected controls. The management of HIV patients experiencing distinct immune reconstitution during effective cART may be improved by understanding more about the specific interplay of key effectors of the innate immune system associated with stem cell mobilization. Once identified, the integrative mechanisms underlying host HIV control should allow us to understand the nature of the immunologic efficacy associated with these rare infected individuals and to eventually exploit these mechanisms to promote a functional HIV cure with strategies including the promotion of antithrombin III coagulation activity or the upregulation of c-Fos-related pro-inflammatory activity.
